# Complete Genome Sequence of *Rhizobium* sp. Strain RCAM05350 from Shulgan-Tash Karst Cave

**DOI:** 10.1128/mra.00569-22

**Published:** 2022-09-26

**Authors:** Anna Sazanova, Vera Safronova, Andrey Belimov, Yuri Gogolev, Elizaveta Chirak, Denis Karlov, Irina Kuznetsova, Lyudmila Kuzmina, Igor Tikhonovich

**Affiliations:** a All-Russia Research Institute for Agricultural Microbiology (ARRIAM), St. Petersburg, Russian Federation; b Kazan Institute of Biochemistry and Biophysics, FRC Kazan Scientific Center of RAS, Kazan, Russian Federation; c Ufa Institute of Biology, Ufa Federal Research Center, RAS, Ufa, Russian Federation; Indiana University, Bloomington

## Abstract

The Shulgan-Tash cave is an extremely interesting object for scientific research, located in the Republic of Bashkortostan (Russia). In this article, we report the complete genome sequence of *Rhizobium* sp. strain RCAM05350 isolated from the “cave silver” biofilms. The sequence was obtained using Oxford Nanopore Technologies MinION.

## ANNOUNCEMENT

The Shulgan-Tash cave (Republic of Bashkortostan, Russia) is an extremely interesting object for scientific research. This cave is developed in the thickness of the lower-Carboniferous gray chemogenic limestones and has specific conditions: a constantly low temperature (from +6 to +7°C), high humidity (100%) and carbon dioxide content, and poor nutrient substrate in the form of lime mud (1). Here, we report the complete genome sequence of *Rhizobium* sp. strain RCAM05350, isolated from the “cave silver” (a biofilm-like microbial community), the study of which is important in terms of their taxonomic structure, as well as metabolic relationships under conditions supporting chemoautotrophy. Aqueous solutions of scrapings from the wall were distributed on R2A agar medium diluted 10 times. The isolate formed visible colonies after 5 days of incubation at 6°C. Genomic DNA was extracted and purified using a DNeasy blood and tissue kit (Qiagen, Germany) according to the recommendation of the manufacturer.

Long-read whole-genome sequencing was performed using a MinION sequencer with the 9.4.1 Rev D flow cell (Oxford Nanopore, England) in the Core Centrum “Genomic Technologies, Proteomics and Cell Biology” at the All-Russia Research Institute for Agricultural Microbiology (ARRIAM). Isolated DNA without size selections was used with the SQK-LSK109 ligation sequencing kit, and the EXP-NBD104 and EXP-NBD114 native barcoding expansion kits were used to prepare the library according to the manufacturer’s instructions. The barcode 7 was used for this genome; 331 million reads were generated at *N*_50_ = 16,780. The reads were basecalled and demultiplexed, and the barcodes were trimmed using the guppy_basecaller (v.5.0.11). The Flye assembler (v.2.8) was used to assemble the Nanopore reads (2). The resulting assembly was corrected 4 times using Racon (v.1.3.2) (3) with the modifiers -m 8 -x -6 -g -8 -w 500, followed by a single polish using the medaka (v.1.4.3) program with default parameters (https://github.com/nanoporetech/medaka). To assess the assembly quality, QUAST (v.5.0.2) was used (4). The assembled genome was shown to present 5 plasmids (289 to 577 kbp) and 1 chromosome with a length of 4,625,629 bp and a total sequence length of 6,492,585 bp, with GC content of 59.7%, and with genome coverage of 46×. A search for genes in the assembled contigs was performed using the NCBI Prokaryotic Genome Annotation Pipeline (PGAP) v.5.3 (5). The circularity of all of the assembled fragments was reported by the Flye assembler and verified by mapping the long reads to the assembled fragments using Minimap2 (6), with the map-ont mapping mode, and inspecting the coverage uniformity; no specific starting point was chosen for the assembled contigs.

The 16S rRNA (*rrs*) gene sequence of *Rhizobium* sp. strain RCAM05350 was compared with those of type strains available from the GenBank database using BLAST analysis at NCBI. This strain is most closely related to the type strain Rhizobium giardinii H152 (98.92% *rrs* similarity, average nucleotide identity of 82.87%), which suggests that it belongs to a new species.

### Data availability.

The complete genome sequence has been deposited in GenBank under accession numbers CP087971, CP087972, CP087973, CP087974, CP087975, and CP087976, corresponding to the sample accession number SAMN23078440, and the raw reads were deposited under accession number SRR21011709.
